# Effects of Vibration on Accelerating Orthodontic Tooth Movement in Clinical and In Vivo Studies: A Systematic Review

**DOI:** 10.3390/dj12080243

**Published:** 2024-07-30

**Authors:** Selma Pascoal, Sofia Oliveira, Margaux Ascione, Jorge Pereira, Óscar Carvalho, Teresa Pinho

**Affiliations:** 1 UNIPRO–Oral Pathology and Rehabilitation Research Unit, University Institute of Health Sciences (IUCS-CESPU), 4585-116 Gandra, Portugal; selma.pascoal@iucs.cespu.pt; 2 Center for Micro-Electro Mechanical Systems (CMEMS), University of Minho, Campus Azurém, 4800-058 Guimarães, Portugal; sofiaoliveira@dem.uminho.pt (S.O.); oscar.carvalho@dem.uminho.pt (Ó.C.); 3 University Institute of Health Sciences (IUCS-CESPU), 4585-116 Gandra, Portugal; margaux.ascione@gmail.com; 4 Faculty of Health Sciences, University Fernando Pessoa, Rua Carlos da Maia, 296, 4200-150 Porto, Portugal; jpereira@ufp.edu.pt

**Keywords:** orthodontics, orthodontic tooth movement, vibration, high-frequency vibration, low-frequency vibration

## Abstract

This systematic review aims to assess the impact of high (>30 Hz) and low (≤30 Hz) frequency vibrations on orthodontic tooth movement (OTM). Several articles were collected through a systematic search in the databases MEDLINE and SCOPUS, following PRISMA methodology and using a PICO question. Relevant information on selected articles was extracted, and the quality of each study was assessed by the quality assessment tools EPHPP, ROBINS-1 and STAIR. Out of 350 articles, 30 were chosen. Low-frequency vibrations did not seem to accelerate OTM with aligners or fixed appliances, despite some positive outcomes in certain studies. Conversely, high-frequency vibrations were linked to increased aligner change, tooth movement, and space closure with fixed appliances. In vivo studies reported favourable results with high-frequency vibrations (60 Hz to 120 Hz), which stimulate bone biomarkers, facilitating alveolar bone remodelling. The results suggest that high-frequency vibration effectively speeds up orthodontic tooth movement, showing promise in both in vivo and clinical studies. Larger-scale research is needed to strengthen its potential in orthodontics.

## 1. Introduction

The so-called “perfect” smile is a result that can be obtained by combining several dental disciplines such as orthodontics, surgery, and prosthetics. Before reaching this famous smile, it is necessary, in most cases, to manage one of the most common problems: malocclusion. This deviation of the closure is, in more than a quarter of teenagers, the cause of aesthetic and functional problems that can have an impact on the quality of life. Therefore, it is important to correct this malocclusion with fixed or removable orthodontic treatment [1]. The duration of an orthodontic treatment is approximately 24 months, varying according to each clinical case [2]. An excessively long treatment will have a negative impact on the patient since it requires control of meals, particular attention to hygiene, regular appointments, and cost. Also, it will have a negative impact on the environment of the tooth, which may cause periodontal diseases or root resorptions [2,3].

Over the years, reducing the duration of orthodontic treatment has become a very important requirement, not only for the patient but also for the orthodontist. In fact, reducing the treatment time can decrease the risk of adverse effects, exposure to associated risks, and cost, and, most importantly, increase patient satisfaction [2,3].

The rate of orthodontic tooth movement (OTM) is the primary factor in reducing treatment time. To shorten treatment time, it is necessary to accelerate OTM and to act on the environment of the teeth, especially the alveolar bone and the periodontal ligament (PDL), which are two structures that ensure stability [2]. Remodelling the alveolar bone and PDL, due to an inflammatory process in response to orthodontic force, promotes cellular and molecular changes. This characterizes the biological process referred to as OTM [4]. Bone cells, namely osteoblasts, and osteoclasts, are the main targets of non-surgical interventions to accelerate OTM [1].

Tooth movement results from the resorption and formation of alveolar bone in response to tension and compression caused by orthodontic forces from braces or aligners. These forces induce pressure on the PDL, triggering vascular changes that release pro-inflammatory molecules, such as interleukin (IL)-1ß, enhancing osteoclast and osteoblast differentiation, and accelerating tooth movement [5,6]. The use of vibration may accelerate OTM by increasing the receptor activator of nuclear factor-kB ligand (RANKL) expression in the periodontal ligament, without additional harm to periodontal tissues, such as root resorption [7]. Direct measurement of RANKL and osteoprotegerin (OPG) levels can provide valuable insights into the osteoclastogenic response to vibration [8]. Vibration could enhance prostaglandin E2 and RANKL effects on human periodontal ligament cells, likely mediated by the cyclooxygenase pathway [9]. It also synergistically boosts osteoclastogenesis and activity via nuclear factor kappa B (NF-κB) activation, leading to alveolar bone resorption and accelerated tooth movement, particularly under continuous static force [10]. Additionally, it could promote differentiation of human PDL cells by increasing type I collagen, runt-related transcription factor 2, and Osterix growth factors [9].

Over the decades, there have been great advances in techniques developed to accelerate the rate of orthodontic tooth movement, both surgical and non-surgical [11,12,13,14].

Vibratory stimuli are an example of a non-surgical method and are most often preferred by patients. There are commercially available devices, such as the AcceleDent^®^ device introduced by MAO in 2006, the aim of which is to accelerate OTM by enhancing bone remodelling through pulsed forces with low-frequency mechanical vibration (LFMV) at 30 Hz, 20 min per day [15]. Another commercial device, but with high-frequency mechanical vibration (HFMV), is the VPro5^®^ device at 120 Hz, 5 min per day [16]. These appliances are easy to use, portable, and can be used in conjunction with fixed appliances and aligners.

Recently, many studies have investigated the effects of vibration. Nevertheless, the methodological heterogeneity and the mostly inconclusive results may cause difficulties in the final positioning of this method, which is still under discussion [3].

Indeed, a comprehensive review of the effectiveness of vibration in accelerating orthodontic tooth movement in both animals and humans is warranted. Animal studies offer better control over the stimulation level, allowing researchers to establish the efficacy of vibration treatment more conclusively. In contrast, clinical studies face challenges in controlling the level of stimulation on individual teeth, potentially leading to inconsistent outcomes. 

Therefore, this work aims to assess the exposure of high (>30 Hz) and low (≦30 Hz) frequency vibration stimuli on orthodontic movement acceleration in patients and animals with an orthodontic force.

## 2. Materials and Methods

To carry out this systematic review, the PRISMA methodology (Preferred Reporting Items for Systematic review and Meta-Analysis Protocols) 2015 checklist was used [17]. This study was submitted and accepted in the PROSPERO database (CRD42024535048).

The “PICO” methodology was used to answer the research question. This methodology is suggested to be more precise and specific in the research. 

The PICO framework for this study is outlined as follows:

- P (Population): Orthodontic patients/animals with an orthodontic force.
- I (Intervention or Exposure): Exposure to high (>30 Hz) or low (≦30 Hz) frequency vibration.
- C (Comparison): Patients/animals who underwent orthodontic treatment with and without vibration stimuli.
- O (Outcomes): OTM by measuring the distance at several points over time intervals.

### 2.1. Research Strategy and Selection Process

The search strategy for this systematic review was conducted using the following MeSH Terms: ((orthodontics OR “orthodontic tooth movement” OR “tooth movement”) AND (vibration OR “high frequency vibration” OR “low frequency vibration”))

The research on MEDLINE and SCOPUS was conducted in April 2024. 

### 2.2. Eligibility Criteria 

The eligibility criteria were as follows.

Inclusion criteria:

- Clinical studies and in vivo studies.
- Vibration combined with orthodontic treatment / orthodontic force.
- Orthodontic treatment with aligners and fixed appliance.

Exclusion criteria:

- Review or meta-analysis, case studies and conference proceedings.
- In vitro studies.
- Articles not in English.
- Combination of vibration with surgical techniques or other stimulation method.
- Numerical simulation studies.

### 2.3. Data Collection and Extraction 

The data extraction process was conducted by two researchers (S.P. and S.O.) who independently reviewed the titles and abstracts of relevant studies. The studies that did not meet the inclusion and exclusion criteria were removed. Any disagreements were resolved through discussion. The full texts of the remaining studies were assessed, and their eligibility criteria were judged. The studies that met the eligibility criteria were included in this systematic review. Data from the selected articles were extracted, and the accuracy of the extraction was verified. The information gathered includes authors’ names, publication year, study type, population, study groups, orthodontic appliances used, location of teeth and desired movement, vibration protocols, results, and conclusions. 

### 2.4. Quality Assessment

The quality assessment of selected studies was carried out. For clinical studies, the tool *Effective Public Health Practice Project* (EPHPP) [18] was applied for randomized controlled trials, while the tool *Risk of Bias In Non-randomized Studies of Interventions* (ROBINS-I) [19] was employed for non-randomized trials. For in vivo studies, the Stroke Therapy Academy Industry Roundtable (STAIR) recommendations [20] were used.

## 3. Results

### 3.1. Search Strategy

**Figure 1:**
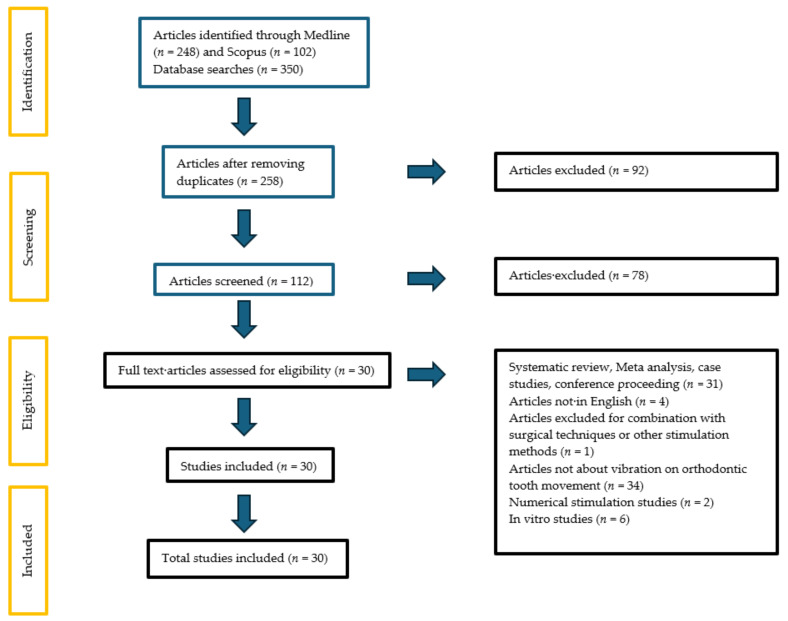
PRISMA flow diagram.

As a result of this research, 350 articles were retrieved, and 92 duplicate records were removed. After reading the titles and abstracts of the remaining 258 studies, 112 relevant articles were selected for full-text reading. After full reading, 78 studies were excluded according to the exclusion criteria. There were 30 studies included in this systematic review. Of the included studies, 25 studies were clinical studies and five were in vivo studies. The PRISMA flow diagram is depicted in Figure 1. 

### 3.2. Quality Assessment

Table 1, Table 2 and Table 3 summarize the quality assessment of the included studies.

Concerning the STAIR quality assessment, regarding sample size, 60% of the studies had a moderate final decision and 40% had a weak final decision. Regarding inclusion and exclusion criteria and allocation concealment, 100% of the studies had a weak final decision. Regarding randomization, 60% of the studies had a weak final decision and 40% had a strong final decision. Regarding the reporting of animals excluded for analysis, only one study was concerned and had a moderate final decision. Regarding blind assessment of outcome, 60% of studies had a weak final decision, 20% a moderate final decision, and 20% a strong final decision. Regarding reporting of potential conflicts of interest and study funding, 80% of studies had a moderate final decision and 20% had a strong final decision. 

Among the 18 randomized clinical trials, the quality of 14 studies was classified as “strong” (78%), while four studies presented a moderate quality assessment (22%).

The quality of seven non-randomized clinical trials was assessed with the ROBINS-I tool. Three studies (43%) were considered to have an overall methodological quality score of “Low”, two studies (29%) presented “some concerns”, and another two studies (29%) were scored as “high”.

### 3.3. In Vivo Studies

The characteristics of the included in vivo studies are presented in Table 4.

The applications of vibration with orthodontic treatment to accelerate tooth movement have been evaluated through animal studies with no positive outcomes, except one study [24] that reported that vibration can be used to improve the rate of tooth movement during the catabolic phase of treatment. 

Levels of stimulation with frequency vibration up to 30 Hz [21,22,24] did not show a significant increase in the rate of OTM. On the other hand, frequencies of 60 Hz, 70 Hz, and 120 Hz had more positive effects, improved OTM [7,23,24], and increased biomarkers of the bone [7,23]. Vibration stimulation was applied between 3 min to 30 min.

Only two studies [7,23] have reported the impact of vibration on root resorption. These studies showed that high-frequency vibration did not affect root resorption.

Out of five animal studies, three were conducted on rats: two [22,23] Wistar (50%) and two [21,24] Sprague Dowley (33%) and one [22] CD1 rats (17%).

The effect of vibration on root resorption was only analyzed in two studies. Both studies evaluated root condition with fixed appliances: one study [38] with HFMV and the other study [40] with LFMV. Both studies reported that root condition was not affected after vibration application.

All studies evaluated maxillary first molar movement to study the acceleration of OTM.

### 3.4. Clinical Studies

The characteristics of the included clinical studies are presented in Table 5.

Out of 25 human studies, 18 [5,8,25,26,27,28,30,32,33,34,35,36,37,38,39,40,47] were randomized controlled trials and seven [16,41,42,43,44,45,46] were non-randomized clinical trials. Six [5,25,28,30,39,41] were split-mouth studies.

This study comprised 1053 patients who needed orthodontic treatment.

Nineteen [5,8,25,26,27,28,30,32,33,34,36,37,38,39,40,41,42,44,47] used fixed appliance as orthodontic appliance, five [16,31,35,45,46] used aligners, and only one [43] used both.

Fourteen [26,31,32,34,35,36,37,39,40,42,43,44,46,47] studies used AcceleDent^®^ as an intervention tool, two [16,45] studies used VPro5^®^, four [5,8,25,30] used an electric toothbrush, and five [27,28,33,38,41] used a customized device. All included daily application. Studies that reported low vibration [8,26,28,31,32,33,34,35,36,37,39,40,42,43,44,46,47] used a frequency of 30 Hz, 20/min day; high-frequency vibration [5,8,16,25,27,30,38,41,45] ranged between 50 and 125 Hz. Only one [8] study explored both types of frequency vibration.

Thirteen [5,8,25,28,30,33,34,36,37,38,39,40,41] studies observed the rate of teeth retraction, four [27,42,44,47] studies observed anterior alignment, three [16,32,43] studies treatment time, four [31,35,45,46] studies aligner replacement; four [5,8,16,26] studies also included biomarkers of bone remodeling, and six [27,31,37,38,40,46] studies also evaluated pain. Seventeen [8,25,26,27,28,31,32,33,34,35,36,37,39,40,41,46,47] out of twenty-five human studies found no significant differences regarding the acceleration of tooth movement with vibration.

Only three studies [42,43,44] with LFMV showed positive results regarding OTM, with increased movement and tooth alignment.

In nine [5,8,16,25,27,30,38,41,45] articles that used HFMV, four [8,25,27,41] did not find significant results regarding OTM acceleration. Two studies [16,45] with a frequency vibration at 120 Hz related a decrease regarding aligners exchange with a decrease in the number of aligners to complete treatment. Regarding the amount of total space closure and canine distalization, one study [30] with vibration frequency at 50 Hz and another [38] at 102 Hz showed increased results.

## 4. Discussion

The professional’s role is to support patients by combining aesthetics with effective orthodontic treatment, and the duration of treatment is becoming increasingly important. As such, in recent years, non-surgical techniques that can be applied at home by the patient with professional recommendations have emerged, such as high or low mechanical vibrations [47]. This alternative technique based on mechanical vibrations is used to increase the rate of orthodontic movement by accelerating periodontal and alveolar bone remodeling [5].

### 4.1. Effects of Supplemental Low-Frequency Vibration on Dental Movement in Humans

Regarding our selected studies, only three reported orthodontic treatment with aligners. Katchooi et al. (2018) [31] and Lombardo et al. (2019) [35] both used the AcceleDent^®^ device with the application according to the manufacturer’s instructions; both concluded that there are no significant effects on the aligner’s regimen times with vibration. However, Bilello et al. (2022) [46], in a similar study did not understand if the acceleration of treatment time could be attributed to the use of AcceleDent^®^ or the change regimen of the aligners. 

Regarding fixed appliances, there is no benefit of vibration in the rate of mandibular space closure, in the duration of treatment and the result [33,36].

Some studies found no statistically significant differences between the groups in the rate of retraction of the maxillary canines [8,28,37,39,40]. In agreement with these results, Telatar et al. (2021) [34] observed in their study in the experimental group (vibration with fixed appliance), the average rate of tooth movement for the lower canines was 1.09 mm per month and 1.24 mm per month for the upper canines, and in the control group (only fixed appliance), the average rate of tooth movement for the lower canines was 1.06 mm per month and 1.06 mm per month for the upper canines; they concluded that canine retraction rates were not different between groups.

Orton-Gibbs (2015) [43] and Bowman (2016) [44] concluded that orthodontic tooth movement and treatment time increased with fixed appliances and AcceleDent^®^. Bowman (2014) [42] found that combining fixed appliances with LFMV from devices like AcceleDent^®^ resulted in a 30% faster treatment duration compared to fixed appliances alone, and another similar study [44] with low frequencies detected a decrease in the number of days to achieve a molar Class I. Orton-Gibbs (2015) [43] reported similar findings, suggesting a significant reduction in treatment duration when using AcceleDent^®^. However, Miles (2018) [32] found no significant difference in treatment duration between the AcceleDent^®^ group and the control group. The existing commercial intraoral vibration device comes with a standard mouthpiece, delivering vibration to teeth primarily through their contact with it. Analysis [48] using finite element methods on this device, under optimal occlusion conditions, reveals an uneven distribution of force over the teeth, with anterior teeth experiencing more stimulation than posterior ones. This force distribution also varies depending on the stiffness of the mouthpiece and individual contact conditions, as each patient’s teeth profile differs in terms of height and angulation. Moreover, if the mouthpiece is not perfectly aligned, some teeth may not receive any stimulation at all. Insufficient dosage and inconsistently targeted delivery can hinder desired biological responses, contributing to the inconsistency in clinical outcomes observed.

ElMotaleb et al. (2024) [40], Taha et al. (2020) [37], and Katchooi et al. (2018) [31] reported pain during orthodontic treatment with low vibration and did not find significant effects on pain reduction.

From a biological point of view, Siriphan et al. (2019) [8] concluded that additional vibratory forces did not improve the rate of tooth movement or the secretion of inflammatory cytokines. Analysis of saliva biomarkers during orthodontic treatment with vibratory stimuli revealed an increase in certain biomarkers like IL-11 and MMP-9 in the control group but not in the group using AcceleDent^®^, suggesting a potential dampening effect on inflammatory response and tooth movement inhibition [26]. Overall, while supplemental vibratory forces show promise in accelerating orthodontic treatment in some studies, others suggest that their effectiveness may vary, depending on factors such as initial tooth irregularity and individual biological responses.

Low-frequency vibration (≦30 Hz) does not appear to accelerate OTM, even though in some studies, it had beneficial effects.

### 4.2. Effects of Supplemental High-Frequency Vibration on Dental Movement in Human Patients 

Few studies [16,45] showed that high-frequency vibration increased orthodontic tooth movement acceleration with aligners. Shipley et al. (2019) [16] observed an increase in aligner change and tooth movement with VPro5^®^ and, consistent with these results, in another study, Shipley (2018) [45], with the same device, observed an increase of 66% in aligner exchanges and an increased number of aligners to complete treatment. 

Some studies [5,30,38] showed that high-frequency vibration increased orthodontic tooth movement acceleration with fixed appliances. Studies by Liao et al. (2017) [30] and Mayama et al. (2022) [38], at 59 Hz and 102 Hz, respectively, demonstrated that the use of supplemental HFMV with fixed appliances led to increased total space closure and canine distalization, indicating that localized high-frequency vibrations can accelerate tooth movement. Nevertheless, it is important to consider the time taken to reach the working wire, as the decision of when to place the next archwire and not waiting for it to be completely passive. In contradiction to these findings, Azeem et al. (2019) [25], Siriphan et al. (2019) [8], and Akbar et al. (2022) [41] did not find statistically significant differences in the amount of canine retraction with fixed appliances. In these studies, toothbrushes were used and the application was only at one point; it may be possible that the direction of vibration application could be an important factor. Another important factor in the application of vibration with fixed appliances is the fact that the arch passes through all the teeth, and if the vibration is applied at a single point, more of the effect is distributed through all the other teeth. As opposed to the treatment with aligners, the vibration is applied without any orthodontic appliance in the mouth, which limits the vibration to only one location without dissipation; the dissipation with fixed appliances also depends on the type of brackets used and the characteristics of the arch.

Two [27,38] studies reported pain during orthodontic treatment with fixed appliances in conjunction with high vibration and did not find significant effects on pain reduction.

Regarding the biological perspective, Leethanakul et al. (2016) [5] studied gingival crevicular fluid (GCF) to assess the response of the tissue to HFMV stimuli from an electric toothbrush. The concentration of the various components of GCF can increase when subjected to vibratory stimuli such as an electric toothbrush. Indeed, the levels of IL-1ß, one of the components of GCF, increase with the additional application of vibratory stimuli during orthodontic treatment. IL-1ß is directly involved in bone resorption since it induces the expression of RANKL in osteoblasts and PDL cells and stimulates osteoclastic precursor differentiation. In this study, IL-1ß levels would be three times higher with additional vibratory force compared to ortho forces alone. This confirms that there is a biological response to the addition of a vibratory stimulus during orthodontic treatment. Therefore, vibration plus orthodontic force increased levels of IL-1ß in the GCF, as well as bone resorption, accelerating the orthodontic movement.

High-frequency vibration consistently appeared to accelerate OTM, although the studies included in this review had a small sample size.

### 4.3. Effects of Supplemental Vibration on Dental Movement in In Vivo Studies

Animal studies demonstrate greater consistency compared to clinical studies due to the ability to exert better control over vibrational force. In these studies, vibrational forces are directly administered to the tooth with adjustable intensity, ensuring a more precise and uniform application of force.

Nishimura et al. (2008) [7] conducted a study applying various resonant frequencies, averaging around 61 Hz, to rats. They observed an average displacement of the first molars at about 0.0014 mm with a velocity of approximately 0.27 mm/second. The study found that tooth movement scale was about 15% higher in the experimental group compared to the control group. Biologically, the experimental group exhibited more multinuclear osteoclasts in the alveolar bone, suggesting a potential link between the number of osteoclasts and the speed of tooth movement. Vibrations stimulated monocyte/macrophage differentiation and increased RANKL expression in PDL osteoclasts and fibroblasts, potentially activating osteoclasts and promoting alveolar bone remodeling.

Takano-Yamamoto et al. (2017) [23] also concluded that vibration resonance had a stimulatory effect on tooth movement speed without negatively impacting tissue. Their study showed that HFMV during dental movement increased the activation of the NF-K B cell signaling pathway in osteocytes, osteoblasts, and osteoclasts. This activation, in turn, enhanced osteoclastogenesis and osteoclast function, leading to accelerated tooth movement through alveolar bone resorption.

Alikhani et al. (2018) [24] investigated the effects of anti-inflammatory drugs on high-frequency acceleration and the role of PDL in orthodontic movement. They found that high-frequency acceleration increased the rate of tooth movement during the catabolic phase and facilitated retention during the anabolic phase of remodeling at the end of treatment. The release of inflammatory mediators in the ligament seemed to play a crucial role in accelerating orthodontic movements.

These studies [7,23,24] showed good results with high-frequency vibration, ranging from 60 Hz to 120 Hz.

In contrast, studies such as Kalajzic et al. (2014) [21] and Yadav et al. (2015) [22] showed differing results regarding the efficacy of LFMV on tooth movement. Kalajzic et al. (2014) [21] noted a smaller distance between molars in the group with fixed appliances with vibration, suggesting that LFMV might inhibit dental movement. Yadav et al. (2015) [22] found that LFMV had no effect on increasing molar displacement in rats and did not increase the number of osteoclasts in teeth not subjected to mechanical forces. They proposed that the alignment of PDL fibers on the tension side during tooth movement might be impaired in rats, affecting osteoclast activation and tooth movement completion. Both studies used low-frequency vibration ranging between 30 Hz and 5 Hz.

### 4.4. Limitations and Strengths of the Study

Some limitations of our study should be highlighted. Not all studies had the same number of patients evaluated, which may distort the results and comparisons between each study. In addition, not all studies used the same vibration device, and, consequently, the same stimulation parameters, which may introduce variability in the results, and, consequently, there is insufficient evidence to support these conclusions. Clinical studies are based on trust between the practitioner and the patient. In fact, the vibratory forces are transmitted to the patient by the patient, which can be problematic as it is influenced by the seriousness of the patient. Only one [26] study reported on patient compliance to the use of the vibration device, concluding that the average compliance rate was 53% and that adherence time decreased throughout treatment. The number of in vivo studies conducted was limited, which weakened the strength of the evidence obtained. Additionally, studies with a high frequency of occurrence included small sample sizes.

Regardless of these limitations, our findings can be used to shape future studies on vibration application in the orthodontic field. This systematic review includes not only animal studies but also clinical studies, providing a broader perspective on the effects of vibration in the OTM, which can aid researchers and clinicians in increasing the clinical performance of this adjunctive therapy. Since vibration is commonly applied at low or high frequency, we have analyzed both modalities, showing that HFMV may be preferred to LFMV in accelerating OTM.

## 5. Conclusions

Vibration is a non-invasive technique that has been gaining attention in recent years. Whether below 30 Hz (LFMV) or above (HFMV), vibration frequencies are applicable in combination with fixed braces but also with aligners, which makes them practical and modern. Our findings showed that in both in vivo and clinical studies, high-frequency mechanical vibration showed promising evidence in accelerating orthodontic tooth movement and may be preferred to low-frequency mechanical vibration. Since studies have shown conflicting results, further research is required in this field, with more precise and rigorous stimulation parameters and larger sample sizes, to better understand the mechanisms of vibration frequencies and their role in OTM. In addition, future studies in the field must investigate optimal vibration protocols (in terms of, e.g., dose, time, the application technique) to address specific types of movement and teeth. The development of customized and programmable stimulation devices designed in a way to specifically stimulate the required direction of movement could also constitute an optimized solution to achieve an effective OTM.

## Figures and Tables

**Table 1:** Quality assessment data for animal studies, using the STAIR preclinical recommendations [20].

	Sample Size Calculation	Inclusion and Exclusion Criteria	Randomization	Allocation Concealment	Reporting of Animals Excluded from Analysis	Blinded Assessment of Outcome	Reporting Potential Conflicts of Interest and Study Funding
Nishimura et al. (2008) [7]	Moderate	Weak	Weak	Weak	NA	Weak	Moderate
Kalajzic et al. (2014) [21]	Moderate	Weak	Weak	Weak	NA	Weak	Moderate
Yadav et al. (2015) [22]	Moderate	Weak	Strong	Weak	NA	Weak	Moderate
Takano-Yamamoto et al. (2017) [23]	Weak	Weak	Strong	Weak	NA	Moderate	Strong
Alikhani et al. (2018) [24]	Weak	Weak	Weak	Weak	Moderate	Strong	Moderate

NA: Not applicable.

**Table 2:** Quality assessment data for clinical trials, using the EPHPP Quality Assessment Tool [18].

	Selection Biais	Study Design	Confounders	Blinding	Data Collection Method	Withdrawals and Dropouts	Final Decision
Leethanakul et al., (2016) [5]	Moderate	Strong	Strong	Moderate	Strong	Strong	Strong
Azeem et al., (2019) [25]	Strong	Strong	Strong	Moderate	Strong	Strong	Strong
Reiss et al., (2020) [26]	Strong	Strong	Strong	Moderate	Strong	Strong	Strong
Miles et al., (2012) [27]	Moderate	Strong	Strong	Moderate	Strong	Strong	Strong
Khera et al., (2022) [28]	Strong	Strong	Strong	Moderate	Strong	Strong	Strong
Woodhouse et al., (2015) [29]	Strong	Strong	Strong	Moderate	Strong	Strong	Strong
Liao et al., (2017) [30]	Strong	Strong	Strong	Weak	Strong	Strong	Moderate
Katchooi et al., (2018) [31]	Strong	Strong	Strong	Strong	Strong	Strong	Strong
Miles, (2018) [32]	Strong	Strong	Strong	Weak	Strong	Strong	Moderate
Siriphan et al., (2019) [8]	Strong	Strong	Strong	Moderate	Strong	Strong	Strong
Kumar et al., (2020) [33]	Strong	Strong	Strong	Moderate	Strong	Strong	Strong
Teletar et al., (2021) [34]	Strong	Strong	Strong	Weak	Strong	Strong	Moderate
Lombardo et al., (2019) [35]	Strong	Strong	Strong	Moderate	Strong	Strong	Strong
DiBiase et al., (2018) [36]	Strong	Strong	Strong	Moderate	Strong	Strong	Strong
Taha et al., (2020) [37]	Strong	Strong	Strong	Weak	Strong	Strong	Moderate
Mayama et al., (2022) [38]	Strong	Strong	Strong	Moderate	Strong	Strong	Strong
Yildiz et al., (2023) [39]	Strong	Strong	Moderate	Moderate	Strong	Strong	Strong
ElMotaleb et al., (2024) [40]	Strong	Strong	Strong	Moderate	Strong	Strong	Strong

**Table 3:** Risk of Bias in Non-randomized Studies of Interventions (ROBINS-I) [19].

	Bias Arising from the Randomization Process	Bias Caused by Deviations from Intended Interventions	Bias Caused by Missing Outcome Data	Bias in Measurement of the Outcome	Bias in Selection of the Reported Results	Overall Bias
Akbar et al., (2022) [41]	Low	Low	Low	Some concerns	Low	Some concerns
Bowman, (2014) [42]	Low	High	High	Low	Low	High
Orton-Gibbs et al., (2015) [43]	High	High	High	High	High	High
Bowman, (2016) [44]	Low	Low	Low	Low	Low	Low
Shipley, (2018) [45]	Low	Low	Low	Low	Low	Low
Shipley et al., (2019) [16]	Low	Some concerns	Low	Low	Low	Some concerns
Bilello et al., (2022) [46]	Low	Low	Low	Low	Low	Low

**Table 4:** Table of results of selected in vivo studies [19].

Author (Year)	Population	Orthodontic Appliance	Treatment Objectives	Vibration Parameters	Results	Conclusion
Nishimura et al. (2008) [7]	N = 12 ♂ 6-week-old Wistar Rats. Groups: - CG: expansive spring to move the upper first molars - EG: additional vibration stimulation applied to the first molars	Fixed appliances: standardized expansive spring made of 0.012-in nickel–titanium wire.	Effects of mechanical stimulation by resonance vibration on tooth movement.	Mean additional vibrational stimulation of 61.02 ± 8.375 Hz, during 8 min on days 0, 7, and 14 during 21 days using an expansive spring	- Mean displacement in resonance was 0.0014 ± 0.002 mm and the average velocity was 0.27 ± 0.018 mm/s. EG: ↑Extent of tooth movement on day 21 (15%) and ↑Osteoclasts - RANKL-positive cells were found in the PDL of both groups. RANKL expression was stronger on the compression side (compared to the tension side). - Root resorption: observed on the root surface of the compression side (both groups).	Application of resonance vibration: might accelerate orthodontic tooth movement. Enhances RANKL expression in the PDL. No additional damage (to periodontal tissues) such as root resorption.
Kalajzic et al. (2014) [21]	N = 26 ♀ Sprague-Dawley rats 7 weeks old Groups: - CG 1: unloaded - CG 2: only vibration - CG 3: only orthodontic spring - EG: orthodontic spring and additional vibrational stimulus	Fixed appliances: nickel–titanium coil spring (25 g of force).	Effect of cyclical force, vibratory, on tooth movement, on the structural integrity of the periodontal ligament, and remodelling of alveolar bone.	Forces at 0.4 N and 30 Hz. 2x/week for 10 min	Statistically significant difference in tooth movement measurements between molars: CG3 (mean of 0.486 ± 0.178 mm) compared to the CG1, CG2, and EG, and when EG (mean of 0.242 ± 0.139 mm) was compared to the CG2 and CG3 groups. It was found at smaller intermolar distances in EG, suggesting that 30 Hz cyclical force inhibited tooth movement. In CG1 and CG2, fewer osteoclasts were found on the alveolar bone and almost no osteoclasts in the periodontal ligament, but a statistical significance in the osteoclast number was found in CG3 (mean: 7.50 ± 1.98) compared to CG1 (1.75 ± 2.06) and CG2 (1.75 ± 1.50). Significant bone volume fraction decrease in CG3 compared to CG1 and CG2. Collagen fibers: tightly thicker morphology in CG2 than CG1. In CG3, collagen fibers were thick and smooth. The EG had a disrupted morphology compared to the CG3.	Cyclical forces significantly inhibited the amount of tooth movement. There was also a disorganization of the collagen fibril structure of the periodontal ligament during dental movement.
Yadav et al. (2015) [22]	N = 64 ♂ CD1 mice 12 weeks old. Groups: - CGs: baseline; no spring + 5 Hz; no spring + 10 Hz; no spring + 20 Hz. - EGs: spring + no vibration; spring + 5 Hz; spring + 10 Hz; spring + 20 Hz.	Nickel-titanium coil springs: 10 g of force, 2 weeks.	To study the effect of low frequencies of vibration on the rate of movement of the teeth, on the BVF, on the tissue density, and on the integrity of the PDL.	LFMV: 5, 10, or 20 Hz. 15 min.	LFMV did not increase the rate of orthodontic tooth movement. Microfocus X-ray computed tomography analysis showed increases in bone volume fractions and tissue densities with applications of LFMV. Sclerostin expression was decreased with 10 and 20 Hz vibrations in both the control and experimental groups. Additionally, the picrosirius staining showed that LFMV helped in maintaining the thickness and integrity of collagen fibers in the periodontal ligament.	There was no significant increase in tooth movement by applying LFMV when compared with the control groups (spring 1 no vibration). LFMV (5, 10, 20 Hz) had no deleterious effect on the integrity of the PDL. However, LFMV maintains the thickness and integrity of the PDL.
Takano- Yamamoto et al. (2017) [23]	N = 70 ♂ 25-week-old Wistar rats (410 g). Groups: - C control group - TM: tooth movement by activated Ni-Ti appliance - V: vibration only - TMV: Ni-Ti appliance and vibration	Continuous static force bent 0.014-inch nickel-titanium wires to the upper right first molar to move it palatally.	To investigate whether the additional application of a vibration device can accelerate orthodontic movement. Searching for the smallest magnitude, the smallest number of applications, and the shortest exposure.	New vibration device with automatic changes: frequency of vibration: 3 gf at 70 Hz, 21 days. EG1 for 3 min, EG2 for 6 min, EG3 for 10 min, EG4 for 30 min.	EG3-Optimal conditions to accelerate orthodontic tooth movement (maxillary molar of adult rats): 3 gf at 70 Hz for 3 min per week (one-week intervals). Optimum magnitude high-frequency vibration, with static force accelerates tooth movement. - Acceleration of experimental tooth movement (by supplemental vibration) did not correlate with the duration of the vibration’s exposure. - Vibration did not produce a directional force that moved the teeth. Thus, vibration alone does not cause tooth movement. - Optimum-magnitude high-frequency vibration with static force did not affect root resorption. - Supplementary vibration with a force synergistically stimulated the activation in osteoblasts, osteoclasts and osteocytes. - During the experimental group: vibration and static force increased the size of the tooth sockets around the periodontal ligament by osteoclastic bone resorption.	The most effective level of supplementary vibration to accelerate tooth movement stimulated by a continuous static force was 3 gf at 70 Hz for 3 min once a week. Furthermore, at this optimum-magnitude, high-frequency vibration could synergistically enhance osteoclastogenesis, leading to alveolar bone resorption and finally, accelerated tooth movement, but only when a static force was continuously applied to the teeth.
Alikhani et al. (2018) [24]	N = 206 ♂ adult Sprague Dawley rats Average weight of 400 g, 120 days of age. Groups: - CG1: animals not received spring nor HFV. - CG2: spring with no activation. - CG3 (orthodontic tooth movement only) - EG: same orthodontic forces and different HFA regimens.	Fixed appliances: Sentalloy closing coils 10 cN or 25 cN of force 1 mm activation	To study the effect of high-frequency acceleration on the rate of movement of teeth and alveolar bone, and to investigate the mechanism by which high-frequency acceleration affects movement.	Acceleration: 0.01 g, 0.05 g or 0.1 g. Frequency: 30 Hz, 60 Hz or 120 Hz. Duration 5 or 10 min.	Increase in acceleration increased the rate of tooth movement Effect of frequency and time on rat of tooth movement is not linear Effects of HFA on the rate of tooth movement are similar to orthodontic forces: Both are PDL-dependent and enhance cytokine release HFA increased osteoclast activity and bone resorption during orthodontic tooth movement	HFA can be used to improve the rate of tooth movement during the catabolic phase of treatment, and can then be used in the anabolic remodelling phase to improve retention. Compared to control groups, 30 Hz demonstrates an increase in the rate of tooth movement Frequencies of 60 Hz and 120 Hz caused an increase in the rate of tooth movement, compared with both control and 30 Hz

Abbreviations: N: Number of animals, CG: Control Group, EG: Experimental Group, PDL: Periodontal Ligament, HFA: High-Frequency Acceleration, RANKL: Receptor of Nuclear Factor Kappa-B Ligand, LFMV: Low-Frequency Mechanical Vibration.

**Table 5:** Table of results of selected clinical studies [19].

Author (Year)	Type of Study	Population	Orthodontic Appliance	Treatment Objectives/Methods	Vibration Parameters	Results	Conclusion
Miles et al., (2012) [27]	Randomized clinical trial	N = 66 Aged 11 and 15 years old. Groups: - CG: without stimulation - EG: Tooth Masseuse^®^	Fixed appliances.	Rate of tooth movement and patient discomfort. Irregularity at 4 time points: start of treatment T0, 5 weeks T1, 8 weeks T2, 10 weeks T3. Level of discomfort at 5 times: after initial brackets and wire placement, 6–8 h after, 1 day after, 3 days after, and 7 days after appliances were placed.	Vibrational frequency of 111 Hz and 0.06 N minimum of 20 min per day.	CG: - Irregularity mean: T0 4.9 mm; T3: 1.6 mm. - Mean irregularity difference: T0–T2: 3.1 mm T0–T3: 3.4 mm EG: - Irregularity mean: T0 6.2 mm; T3: 2.1 mm. - Mean irregularity difference: T0–T3: 4.0 mm	No significant differences in the levels of irregularity or pain between the two groups.
Bowman (2014) [42]	Non-randomized clinical trial	N = 117 ♂47 ♀70 Groups: - EG: AcceleDent^®^ vibration group - CG1: Fixed appliance only - CG2: Patients who were treated prior to the initiation of a separate pro- spective examination of the effects of vibration on molar distalization	Fixed appliances.	Effects of vibrational device on the time required for lower arch levelling and alignment on Class II non-extraction patients who underwent upper molar distalization.	AcceleDent^®^ device. Low-frequency vibration 30 Hz, 0.25 N, for 20 min per day.	- Mean time period required to attain alignment of the lower arch dentition was shorter in the EG groups than in the CGs (respectively 93 < 120 < 131 days). - The following arch wire (0.017” × 0.025”) was placed in the EG group earlier than in the CGs: 27 days earlier than CG1 patients and 38 days earlier than in the CG2 group (respectively, 29% and 40% faster). - Levelling took approximately 5 months in EG and 7 months in CGs.	AcceleDent^®^: ↓amount of time needed to achieve dental alignment and levelling in Class II non extraction patients. ↑30% tooth movement during levelling of the lower arch dentition.
Woodhouse et al., (2015) [29]	Randomized controlled trial.	N = 81 ♂ 40 ♀41 Mean age of 14.1 years. Groups: - EG 1: fixed appliance treatment with AcceleDent^®^ - CG 1: fixed appliance treatment with identical non-functional device (sham) - CG 2: fixed appliance only.	Fixed appliances.	Effect of additional vibration force on the rate of orthodontic alignment of teeth with fixed appliances.	AcceleDent^®^ device. Low-frequency vibration 30 Hz, 0.25 N, for 20 min per day.	- Mean irregularity index at baseline for the total sample: 8.5 mm (±3.8) with no significant difference among groups. - Mean irregularity index at initial alignment for the total sample: 2.7 mm (±2.8) with no significant difference among groups. - Mean time from initial to final alignment: 150 days (±62.5). - Mean time from baseline to final alignment: 209 days (±65). There were no significant differences among groups. - For each mm of irregularity, initial rate, initial rate of alignment increased by 0.01 mm/day, and overall rate increased by 0.004 mm/day.	No evidence that the additional vibratory force increases the rate of initial tooth movement or reduces the time required to achieve final alignment.
Orton-Gibbs et al., (2015) [43]	Non-randomized clinical trial	N = 117 Mean age of 31 years. 76% adults ♀66% ♂ 44% Groups: Group with fixed appliance: - EG: fixed appliances and AcceleDent^®^ - CG: fixed appliances only Group with aligners: - EG: aligners and AcceleDent^®^ - CG: aligners only	Ceramic brackets (N = 52) Metal brackets (N = 19) Lingual brackets (N = 19) Clear aligners (N = 16) Removable expansion appliances (N = 11)	Study the processing time with the AcceleDent^®^ experiment.	AcceleDent^®^ device. Low-frequency vibration 30 Hz, 0.25 N, for 20 min per day.	Group with fixed appliances: In the group with patients with fixed appliances and using AcceleDent^®^, treatment took an average 12.4 months: 38.2% faster than the predicted time (20.0 months). In the group with patients without AcceleDent^®^, predicted treatment time was accurate to within an average of 1.6 months. Predicted times were between 18 and 24 months. Group with aligners: - Patients with aligners and AcceleDent^®^ had to change aligners as they became passive (7 to 10 days): treatment time was an average 37.2% faster than the conservative estimate, with a range of 5–55% faster.	AcceleDent^®^ in patients with fixed appliances or aligners ↓treatment time.
Bowman (2016) [44]	Non-randomized clinical trial	N = 30 Mean age: EG 13.1 years, CG 2.9 years ♂13 ♀17 in each group. Groups: - EG: AcceleDent^®^ throughout orthodontic treatment - CG: orthodontic treatment only	Fixed appliances	Study the effects of vibration on molar distalization.	AcceleDent^®^ device. Low-frequency vibration 30 Hz, 0.25 N, for 20 min per day.	- Significant difference between EG and CG with respect to 2nd-molar eruption status. - No statistically significant differences between groups with respect to upper 1st molar tipping, intrusion, crown distalization, number of days required to achieve a super-Class I molar relationship. - EG 27% greater rate of crown movement (1.1 mm/month vs. 0.9 mm/month). Also, 71% more movement of the molar root apex (2.9 mm/month vs. 1.7 mm/month).	↑30% of the mandibular arch levelling rate ↑almost 3 × the tooth movement per month typically reported of 1 mm in the maxillary arch ↑150–200% reduction in time required.
Leethanakul et al., (2016) [5]	Randomized controlled trial.	N = 15 Mean age 22.9 years. Groups: - EG: one side with light force of 60 g applied to the canine for three months + vibratory stimuli - CG: Contralateral canine: only orthodontic force.	Fixed appliances.	Effects of vibratory stimuli applied with an electric toothbrush on interleukin IL-1 secretion during upper canine distalization.	Electric toothbrush Battery-powered Colgate^®^ Motion-Multi Action. 125 Hz. 15 min per day for 2 months. Time points: T0 before starting canine retraction T1 after canine retraction for one month T2 after canine retraction for two months without and with vibration T3 after canine retraction for three months without and with vibration.	- Patterns of fluctuation in the GCF’s volumes were similar at the pressure and tension sites of experimental and control canine. - IL-1β level at the pressure site of control teeth: higher for experimental teeth than for control teeth. IL-1β level for experimental teeth, after one month of vibration, did not reduce at T2 and stayed like levels at T1 and then elevated significantly at T3. - IL-1β level at the tension site of control teeth: no fluctuations observed. - IL-1β level at the tension site of experimental teeth: increased after vibration at T2 and T3 time points. At T3, a significant difference in the IL-1β levels were observed between the tension sites of experimental teeth. - IL-1β levels of experimental teeth were higher at the pressure site after retraction and after one month of vibration. IL-1β levels of control teeth were higher at the pressure site after retraction. - Within T0 and T1, the amount of canine movement was equal for experimental and control teeth. At T2 and T3, the amount was higher for experimental teeth compared to control teeth. After T2, the amount of movement doubled for the experimental teeth. - Within T2 and T3, the amount of tooth movement reduced for the experimental teeth but remained higher than control teeth.	Vibratory stimuli, in combination with orthodontic force: ↑secretion of IL-1β in GCF ↑bone resorption activity and acceleration of tooth movement.
Liao et al., (2017) [30]	Randomized controlled trial.	N = 13 patients Mean age:13.6 years. Patients were randomly assigned a vibration and a non-vibration side on the buccal surface of upper canine.	Orthodontic fixed appliances: brackets.	Examine the biomechanics of orthodontic movements when subjected to a single tooth vibration with a conventional orthodontic force. Distal retraction of maxillary canines.	Oral B^®^ Hamming Bird Vibrating device with a frequency of 50 Hz and a force of 0.2 N, 10 min per day.	- Amount of total space closure on canine distalization is higher with vibration (compared to non-vibration). - Harmonic vibration in conjunction with orthodontic force: amplify the VHS of the PDL. - 50 Hz vibration: increased the VHS of canine PDL (9.2% for the mesio-distally and 10.8% for the linguo-buccally). - Highest amplification induced by the vibration of 50 Hz was recorded with lateral incisor with mesio-distal vibration and 1st premolar with linguo-buccally vibration. - General enlargements of tissue responses: ranging from 7.3% to 13.5% - Higher amplifications for 1st and 2nd premolars and canine for linguo-buccal vibration.	The amount of total space closure ↑with vibration and the amount of distalization of the canine ↑on the vibration side.
Lombardo et al., (2019) [35]	Randomized controlled trial.	N = 45 Mean age of 27.1 ♀ 25 ♂ 20 Groups: - CG: Group A: 15 patients, conventional protocol with aligners replaced every 14 days. - EG: Group B: 15 patients with aligners substituted every 14 days + AcceleDent^®^ - EG: Group C: 15 patients with aligners substituted every 7 days + AcceleDent^®^	Aligners	Differences in the accuracy of tooth movement in patients who are treated with a low-frequency vibratory aligner AND reducing OR reducing the interval of aligner replacement compared to a conventional protocol.	AcceleDent^®^ device. Low-frequency vibration 30 Hz, 0.25 N, for 20 min per day.	- Rotation of the upper incisors: group B is significantly more accurate than group A. (0.72 > 0.62) - Vestibulo-lingual tipping of the upper canines: group B significantly more accurate than group C. (0.67 > 0.54) - Mésio-distal tipping of the upper canines: group B significantly more accurate than group C. (0.65 > 0.49) - Vestibulo-lingual tipping of the upper molars: group B significantly more accurate than group C. (0.71 > 0.55)	No difference in accuracy between the aligners (replacing every 7 days) with low-frequency and aligners (replacing every 14 days) without vibration.
Katchooi et al., (2018) [31]	Randomized controlled trial.	N = 27 Mean age of 33 years. ♂ 12 ♀ 15 Groups: - EG: N = 14 AcceleDent^®^ and aligners - CG: N = 13 Sham AcceleDent^®^ and aligners	Aligners	Effects of AcceleDent^®^ when used in conjunction with Invisalign^®^ (clear aligners).	AcceleDent^®^ device. Low-frequency vibration 30 Hz, 0.25 N, for 20 min per day.	- Both groups were compliant with usage of AcceleDent^®^ and there was no significant difference between completers and non-completers of the 1-week change regimen. - No significant difference in rates between EG and the CG (77% and 85% respectively). - T-tests indicated no difference in the irregularity index between the EG and the CG. - Pain levels lower in the group with active AcceleDent^®^. But the differences were significantly different at day 3 of the first set of aligners.	No evidence that AcceleDent^®^ completes a series of alignments (weekly change) or final alignment obtained in the 27 patients. No significant effects on pain reduction or quality of life when AcceleDent^®^ is used with Invisalign^®^
DiBiase et al., (2018) [36]	Randomized clinical trial.	N = 61 Mean age 13.9 ♂ 30 ♀ 31 Groups: - EG: AcceleDent^®^ With fixed appliance - CG: Fixed appliances only	Fixed appliances.	Study the effect of an additional vibratory force on space closure and the results on patients with fixed appliances	AcceleDent^®^ device. Low-frequency vibration 30 Hz, 0.25 N, for 20 min per day.	- Initial rate of mandibular arch space closure: median of 0.89 mm/month with no significant differences among groups. - No significant differences among groups for any secondary outcomes, -overall treatment duration (median 18.57 months) - overall rate of mandibular arch space closure (median 0.74 mm/month) - number of visits (median 12 visits) - number of breakages (median 2 breakages) - absolute (median 28 points) - % of improvement in PAR score (median 90.0%) - final PAR score (3 points).	There is no benefit with vibration in the rate of mandibular space closure, in the duration of treatment and in the result. Furthermore, the use of additional vibratory forces with fixed devices is not associated with increased device breakage
Miles, (2018) [32]	Randomized controlled trial.	N = 40 Mean age of 12.8 years. ♀ 26 ♂ 14 Groups: - EG: AcceleDent Aura^®^ with fixed appliances - CG: fixed appliances only	Fixed appliances.	Difference in time to reach the working wire stage with the AcceleDent^®^ device compared to the control group.	AcceleDent^®^ device. Low-frequency vibration 30 Hz, 0.25 N, for 20 min per day.	- Upper arch: AcceleDent^®^ group took a median 139 days and the control group took 132 days. - Lower arch: AcceleDent^®^ group took a median 143 days and the control group took 139 days.	In the upper arch, the difference in treatment time for the AcceleDent^®^ group and the control group is not significant. The same is true for the lower arch.
Shipley, (2018) [45]	Non-randomized clinical trial	N = 16 EG: 27.6 years; CG age: 18.9 years. ♀ 11 ♂ 5 Groups: - EG with aligner treatment + use of the HFA device. Exchanged aligners every 5 days. - CG: aligner treatment, exchange aligners every 14 days.	Aligners	Effect of an HFA on clear aligners exchange intervals + treatment time needed to achieve prescribed tooth movements.	VPro5^®^ device 120 Hz, 5 min per day.	- Lower and upper crowding were at 0.0 mm for all patients in EG and CG at post-treatment. - Prescribed number of aligners was not significantly different between groups (14 days), but the number used in EG was lower than among controls (approximately 5 days and 14 respectively). This equates to a 66% reduction. - Estimated treatment durations between groups did not differ significantly. Treatment duration for EG was significantly shorter than CG. EG duration was significantly shorter than estimated and CG duration was significantly longer than estimated.	VPro5^®^: ↑66% aligners exchange ↓number of aligners to complete treatment
Azeem et al., (2019) [25]	Randomized controlled trial.	N = 28 Mean age of 20.8 years. ♀ 18 ♂ 10 Groups: - EG: vibration-side with a light force of 100 g applied to the canine for 90 days + vibratory stimuli - CG: non-vibration-side with only orthodontic force applied to the canine	Fixed appliances.	Effect of an electric toothbrush (vibratory stimuli) on the speed of orthodontic tooth movement during maxillary canine retraction	Orthodontic brush head Oral-B Triumph^®^ 125 Hz. 20 min per day.	- First month of canine retraction: amount of canine movement equal for the vibration side and non-vibration side. - Second and third months: also similar for the canines on the vibration side and non-vibration side. - Plaque accumulation: minimal during the study. No statistically significant difference between the vibration and the non-vibration sides. - No reported discomfort.	Application of supplemental vibratory stimuli (electric toothbrush) in combination with light orthodontic force do not accelerate tooth movement.
Siriphan et al., (2019) [8]	Randomized controlled trial.	N = 60 Mean age 21.5 years ♀ 47 ♂ 13 Groups: - EG 1: canine combined with 60 cN continuous distalization force and 30 Hz vibration - EG 2: canine combined with 60 cN continuous distalization force and 60 Hz vibration - CG: canine combined with 60 cN continuous distalization force only	Fixed appliances.	Effects of a vibratory stimulus on canine distalization and OPG and RANKL secretion.	30 Hz vibration or 60 Hz vibration produced by modified electric toothbrushes, for 20 min per day Timepoints: T1: Before initiation of distalization. T2: 24 h after initiation of distalization. T3: 48 after initiation of distalization. T4: 7 days after initiation of distalization.	- Compression side in the group control: RANKL is significantly different between time points. - RANKL at T1 is significantly lower than T2, T3, or T4 - RANKL, OPG, and RANKL/OPG ratio: not significantly different within or between groups. - Rates of canine movement: not significantly different between the 30 Hz (0.82 mm/month), 60 Hz (0.87 mm/month), and control groups (0.83 mm/month). - Rates of molar movement and tipping and canine rotation and tipping: not significantly different between groups.	30 Hz and 60 Hz vibrations have no additive effect on rate of canine distalization, OPG, and RANKL secretion or RANKL/OPG ratio.
Shipley et al., (2019) [16]	Non-randomized clinical trial	N = 30 ♀ 19 ♂ 11 Groups: - EG: VPro5^®^ with aligners - CG: only aligners	Aligners	Effects of HFV on tooth movement and post-treatment bone density at initiation of retention.	VPro5^®^ device 120 Hz, 5 min per day.	- Patients with adjunctive HFV exchanged aligners faster than the CG. They also finished the treatment faster. EG average clear aligners exchange interval was 5.2 ± 2.2 days whereas for the control group it was 8.7 ± 1.2 days. EG average total treatment time was 135 ± 27 days whereas for the control group it was 252 ± 59 days. A substantial increase in bone density in the alveolar bone around teeth in the maxilla and mandible was observed in the EG, but not in the CG at the beginning of the retention phase.	VPro5^®^ ↑aligner change ↑tooth movement ↑bone density
Kumar et al., (2020) [33]		N = 65 Mean age of 17.1 years. ♀ 35 ♂ 30 Groups: - EG 1: passive self-ligating brackets treated + low-frequency vibrations - EG 2: Conventional MBT brackets + low-frequency vibrations - CG: conventional MBT brackets only	Fixed appliances: conventional MBT brackets and passive self-ligating brakets	Effectiveness of frequent low-frequency vibration on orthodontic movements in patients with passive self-ligating brackets and conventional brackets.	30 Hz vibration, 20 min per day	- Rate of space closure in EG 1: 0.61 mm/month (maxillary right side), 0.61 mm/month (maxillary left side), 0.51 mm/month (mandibular right side), 0.51 mm/month (mandibular left side). - Rate of space closure in EG 2: 0.54 mm/month (maxillary right side), 0.54 mm/month (maxillary left side), 0.46 mm/month (mandibular right side), 0.46 mm/month (mandibular left side). - Rate of space closure in CG: 0.57 mm/month (maxillary right side), 0.61 mm/month (maxillary left side), 0.53 mm/month (mandibular right side), 0.53 mm/month (mandibular left side).	Low frequency does not increase in a statistically significant way the orthodontic tooth movement.
Taha et al., (2020) [37]	Randomized- controlled trial	N = 21 Mean age of 15.48 years. ♀ 14 ♂ 7 Groups: - EG: mechanical vibration device with fixed appliance - CG: fixed appliance only	Fixed Appliances	Retraction of maxillary canines under the effect of a mechanical vibration simulation, and perception of pain in patients undergoing complete orthodontic treatment with extraction.	AcceleDent^®^ device. Low-frequency vibration 30 Hz, 0.25 N, for 20 min per day. Timepoints: T0: day of initial canine retraction T1: 4 weeks after initiation canine retraction T2: 8 weeks after initiation canine retraction T3: 12 weeks after initiation canine retraction	Amount of tooth movement in CG vs. EG: 1.12 ± 0.22 mm vs. 1.39 ± 0.36 mm at T1. 2.59 ± 0.38 mm vs. 2.49 ± 0.76 mm at T2. 3.54 ± 0.24 mm vs. 3.37 ± 1.38 mm at T3. Rate of tooth movement: Overall tooth movement rate of 1.21 ± 0.32 mm/month for CG and 1.12 ± 0.20 mm/month for EG. 1.12 ± 0.22 mm/month (control) vs. 1.39 ± 0.36 mm/month (experimental) at T1. 1.47 ± 0.37 mm/month (control) vs. 0.93 ± 0.59 mm/month (experimental) at T2. 1.01 ± 0.31 mm/month (control) vs. 1.05 ± 0.71 mm/month (experimental) at T3. Level of pain: - Slightly higher in the EG on the 1st day. - Day 2: similar in the two groups. - Day 3: started to stay higher in the EG until day 6 (started to reduce to similar levels).	There were no statistically significant differences between the groups in the rate of retraction of the maxillary canines or in the pain perceived with the use of the mechanical vibration stimulation device.
Reiss et al., (2020) [26]	Randomized controlled trial	N = 40 Mean age 20.4 years. ♀ 20 ♂ 20 Groups: - EG: supplemental use of vibrational device with fixed appliances - CG: fixed appliances only	Fixed Appliances	Effect of additional vibratory force, in patients with fixed appliances, on biomarkers of bone remodelling, to study the RMAA and to study compliance with a vibrating appliance.	AcceleDent^®^ device. Low-frequency vibration 30 Hz, 0.25 N, for 20 min per day. Timepoints: T0: baseline measurements T1: 4–6 weeks later T2: 10–12 weeks later T3: 15–18 weeks later	Mean first quartile and third quartile of irregularity and biomarkers including RANK/OPG ratio in the AcceleDent^®^ and control group: - 7.24 at T0 vs. 8.96 at T0 - 4.26 at T1 vs. 5.24 at T1 - 2.33 at T2 vs. 2.96 at T2 - 0.97 at T3 vs. 1.22 at T3 The change did not significantly differ between groups. In the CG: Biomarkers level increased (remained similar in the EG).	No difference in the changes in salivary biomarkers of bone remodelling and no correlation was found between changes in irregularity and biomarker level. No association between RMAA and compliance with additional vibratory force.
Telatar et al., (2021) [34]	Randomized, controlled trial.	N = 20 ♀ 10 ♂ 10 Groups: - EG: AcceleDent^®^ device with fixed appliances - CG: fixed appliances only	Fixed appliances	Evaluate the application of vibrational forces on the rate of canine distalization.	AcceleDent^®^ device. Low-frequency vibration 30 Hz, 0.25 N, for 20 min per day.	In the EG, the average rate of tooth movement for the lower canines was 1.09 mm per month and 1.24 mm per month for the upper canines. In the CG, the average rate of tooth movement for the lower canines was 1.06 mm per month and 1.06 mm per month for the upper canines. Canine retraction rates were not different between groups.	No statistical difference between groups in the rate of canine retraction.
Mayama et al., (2022) [38]	Randomized controlled trial.	N = 25 Mean age 20.2 years. ♀ 21 ♂ 4 Groups: - EG: vibration with fixed appliance on one side of the upper arch - CG: fixed appliance on the other side of the upper arch	Fixed appliances.	Evaluate the effect and safety of supplementary vibrations on orthodontic movements.	Frequency of vibration 102.2 ± 2.6 Hz Force of 5.2 ± 0.5 g. 3 min per day.	- Mean amount of canine movement at each visit: 0.89 mm ± 0.55 mm in the CG group and 1.21 mm ± 0.60 mm in EG (significant difference). - Number of estimated visits for space closure: 6.38 ± 3.10 in CG and 4.61 ± 2.15 in EG. Number of visits in the EG was significantly less than in the CG. The difference between the two groups was 1.77 ± 4.65. - No patients complained of any pain during 3 min of vibration (supplemental) with static orthodontic force. - Evaluation of the VAS: no significant difference between the two groups. - Crown root ratio at the start of canine retraction was 0.59 ± 0.02 in the CG and 0.58 ± 0.02 in the EG. - Crown root ratio at the end of canine retraction was 0.59 ± 0.02 in the CG and 0.59 ± 0.02 in the EG. No significant difference was observed.	No statistically significant differences in pain, discomfort, and root resorption. Static orthodontic force with additional vibration ↑tooth movement (canine retraction), ↓number of visits
Akbar et al., (2022) [41]	Non-randomized clinical trial	N = 30 ♀ 20 ♂ 10 Groups: - EG: left side in the upper arch with vibration with fixed appliance - CG: right side in the upper arch only with fixed appliance	Fixed appliances.	Effect of localized vibration on the degree of retraction of the canines and evaluate the loss of anchorage.	Vibration by Oral B^®^ electric toothbrush 240 Hz. 15 min per day.	- No discomfort was reported. - After 12 weeks of canine retraction: no difference in the amount of canine retraction between the groups. - Anchorage loss in the form of molar mesialization: present in both groups but with a difference insignificant. - Difference between the anchorage loss in the form of maxillary canine and 1sr molar rotation: statistically insignificant.	No statistically significant differences in the amount of canine movement; no difference in anchorage loss.
Bilello et al., (2022) [46]	Non-randomized Clinical trial	N = 20 Mean age 35 years. ♀ 75% Groups: - EG: 7-day aligner change regimen + AcceleDent^®^ - CG: 14-day aligner change regimen, no supplemental vibration.	Aligners.	Investigate the effectiveness of AcceleDent^®^ device when used during a clear aligner treatment.	AcceleDent^®^ device. Low-frequency vibration 30 Hz, 0.25 N, for 20 min per day.	EG needed 41.1 ± 22.4 aligners on average. CG needed 33.1 ± 15.5 aligners. Difference between the two groups was not statistically significant. Treatment duration EG: 366 ± 187.4 days. Treatment duration CG: 509.3 ± 243.5 days. To complete the treatment, EG wore each aligner 9.0 ± 1 days and CG wore each aligner 15.4 ± 1.2 days. There is an average difference of 6.4 days per aligners, the difference is statistically significant. Pain perception: EG reported a VAS mean value of 2.4 ± 1 and CG a VAS mean value of 4.4 ± 1.4.	Using AcceleDent^®^ device during a clear aligner treatment led to a successful, fast, and comfortable treatment, with reduction in pain perception, although they did not understand if the acceleration of treatment time could be attributed to the use of AcceleDent^®^ or to the change regimen of the aligners.
Khera et al., (2022) [28]	Randomized controlled trial	N = 30 Aged between 18–25 years. Groups: - EG: Vibration side with fixed appliances - CG: fixed appliances only	Fixed appliances.	Investigate the effect of low-frequency vibration on the rate of canine retraction.	Customized vibratory device. Frequency of 30 Hz and force of 0.25 N. 20 min per day. Timepoints: T0: baseline. T1: 1st month. T2: 2nd month. T3: 3rd month. T4: 4th month.	This study showed a statistically non-significant difference between groups in the rate of canine retraction.	Low-frequency vibratory stimulation of 30 Hz with fixed orthodontic treatment did not significantly accelerate the rate of canine retraction.
Yildiz et al., (2023) [39]	Randomized controlled trial	N = 24 ♀ 8 ♂ 16 Mean age 15.07 Groups: CG: orthodontic force (split-mouth: one side with an activation only force, and the other side with an intermittent activation-deactivation-activation force) EG: orthodontic force (split-mouth) with AcceleDent^®^ device	Fixed appliance with Hycon device	To achieve a synergistic effect between Hycon and AcceleDent^®^ on the accelerating orthodontic tooth movement with the least possible root resorption.	AcceleDent^®^ device. Low-frequency vibration 30 Hz, 0.25 N, for 20 min per day.	Intermittent force provided more effective and rapid canine distalization (statistically significant difference on both groups with or without vibration). Comparing the same activating protocol with vibration on the speed of tooth movement the results showed no significant difference.	The intermittent force was very effective in closing spaces. Vibration did not significantly affect orthodontic tooth movement rate.
ElMotaleb et al., (2024) [40]	Randomized controlled trial	N = 32 (64 canines) ♀ Age 15–21 years Groups: CG: fixed appliance only EG: fixed appliance and vibration device	Fixed appliance	Investigate the effectiveness of AcceleDent^®^ vibrating device on the rate of canine retraction.	AcceleDent^®^ device. Low-frequency vibration 30 Hz, 0.25 N, for 20 min per day. 4 months	No statistically significant difference between both groups regarding the total distance of canine retraction. No statistically significant difference between both groups regarding the rate of canine retraction per month. No statistically significant difference between both groups regarding pain level. Root condition was the same for both groups.	No evidence that AcceleDent^®^ had any effect on acceleration of tooth movement. Pain level could not be reduced. Root condition was not affected.

Abbreviations: N: Number of patients, CG: Control Group, EG: Experimental Group, IL: Interleukin, GCF: Crevicular Gingival Fluid, PDL: Periodontal Ligament, VHS: Volume-average Hydrostatic Stress, HFV: High-Frequency Vibration, RANKL: Receptor of Nuclear Factor Kappa-B Ligand, OPG: Osteoprotegerin, RMAA: Rate of Mandibular Anterior Alignment.

## Data Availability

The data supporting the findings of this review can be found within this article, as the authors used published data sets.
